# Mapping the developmental path for Parkinson’s disease therapeutics

**DOI:** 10.1038/s41531-025-01154-1

**Published:** 2025-11-07

**Authors:** Neel T. Dhruv, Sarah Robinson Schwartz, Christine Swanson-Fischer, Hyun Joo Cho, Rebekah Corlew, Lyn Jakeman, Lauren A. Laboissonniere, Rebecca Price, Shireen Sarraf, Beth-Anne Sieber, Christine Torborg, Carl Wonders, Alice Chen-Plotkin, William J. Martin, Amir P. Tamiz

**Affiliations:** 1 National Institute of Neurological Disorders and Stroke, Bethesda, MD USA; 2 Department of Neurology, Perelman School of Medicine, University of Pennsylvania, Philadelphia, PA USA; 3 Johnson & Johnson, Inc., San Diego, CA USA

**Keywords:** Parkinson's disease, Neurodegeneration, Target validation, Biomarkers

## Abstract

The National Institute of Neurological Disorders and Stroke (NINDS) held an open workshop on April 23 and 24, 2024 to evaluate the gap between the current understanding of Parkinson’s disease (PD) and the development of therapeutics for treating PD. Representatives from key stakeholder groups discussed strategies for leveraging research to bridge this gap. Sessions focused on PD heterogeneity, target validation, development of tools and resources to facilitate therapeutics development, biomarker discovery and use, similarities and differences with PD-adjacent neurodegenerative diseases, and best practices for accelerating the therapeutics development process. Here are some of the main takeaways from the workshop.

## Introduction

Parkinson’s disease (PD) is a progressive neurodegenerative disease manifesting in a wide array of motor and non-motor symptoms. The disease affects about 1 million individuals in the United States of America, and several million people worldwide, thereby posing a substantial public health burden and concomitant economic cost^1,2^. In 2019, the National Institute of Neurological Disorders and Stroke (NINDS) convened a workshop to identify strategies to accelerate clinical advancement of disease-modifying therapies for PD^3^. A central outcome of that workshop emphasized critical steps in the preclinical phase of therapeutics development process, with a particular emphasis on selecting molecular targets and developing robust biomarkers for disease diagnosis and progression^4^.

Despite the substantial public and private investment in therapeutics development for the PD community, a separation persists between our understanding of the disease and the successful translation of this knowledge into effective treatments targeting its underlying pathophysiological mechanisms. To address this challenge, NINDS recently convened a hybrid workshop (https://www.ninds.nih.gov/news-events/events/hybrid-workshop-advances-therapeutics-development-parkinsons-disease) with the primary objectives to (1) delineate the critical gaps in our current understanding and (2) establish a collaborative framework for bridging those gaps. The workshop agenda is included in the Supplementary Information. This effort brought together a diverse community of stakeholders, including academic researchers, industry partners, non-profit organizations, and people with lived experience (PWLE) of PD to discuss the relevant concerns and considerations on how to drive the process forward (Box 1). The result is a roadmap of future research and therapeutic development efforts that the community may find useful.

Box 1 Breakout sessions As part of the workshop, attendees split into one of six breakout sessions, each tasked with identifying key knowledge gaps and challenges in PD research that hinder therapeutics development. Participants also explored opportunities and potential solutions to overcome these barriers. Discussions were organized under three overarching themes: (1) key points of resonance from the workshop; (2) strategies to accelerate translation; (3) approaches to generate new therapeutics.

***Key points of resonance from the workshop***

- Industry relies on foundational research conducted in university and government laboratories to generate insights into disease mechanisms to inform novel therapeutic target identification. However, there are often legal and logistical challenges in establishing collaborations between partners.
- Research programs can promote and facilitate collaboration between academia, industry, and regulatory agencies. Care should be taken to ensure transparent agreements to govern the sharing of reagents, biospecimens, and data from both preclinical and clinical studies.
- Improved mechanisms for data sharing across studies, particularly between academia and industry researchers, are needed to accelerate discovery.
- A robust research infrastructure can incentivize studies which recapitulate and validate key findings. This infrastructure would ideally incorporate centralized data repositories to facilitate data sharing and provide analytical tools for handling large, complex datasets.
- A comprehensive understanding of PD heterogeneity is critical for advancing therapeutic development.
- Patient subtyping based on molecular and multimodal profiles may enhance precision medicine approaches in PD.
- Engagement with PWLEs is crucial for patient-centered preclinical research and informing clinical trial design.
- The PD community could benefit from a “Patient’s Bill of Rights”, which would outline rights related to biomarker test results, including the choice to receive or withhold such information, and the autonomy to decide on a therapeutic course.
- PWLE discussants encouraged expanding PWLE representation in clinical trial review panels and patient advisory councils at the funding stage.
- Communication within and between a patient’s care team is necessary to help the patient get the care they need.

***Strategies to accelerate translation***

- Leveraging unique genetic signatures can help to identify causal biology, putative drug targets, and disease-specific biomarkers.
- The success of amyloid PET imaging in tracking disease progression for AD underscores the need for a comparable biomarker in PD. While no single biomarker is likely sufficient to characterize PD progression, composite biomarker signatures may prove effective.
- Expanding biomarker discovery beyond αSyn and other pathological mechanisms to include functional endpoints, such as digital health technologies, to track motor and non-motor symptoms, may provide a more comprehensive assessment of disease progression. Additionally, the development of “companion” biomarkers to assess target engagement in therapeutic trials is of key importance.
- Optimizing data curation strategies is essential for the efficient management of large PD databases.
- Animal models remain useful tools for pharmacokinetics, pharmacodynamics, and toxicology studies.
- Human iPSC-derived neuronal models provide critical insights into target engagement and therapeutic mechanisms.
- The development of organoid models incorporating diverse cell types beyond neurons will enhance the translational relevance of in vitro studies.

***Approaches to generate new therapeutics***

- Given the complexity of PD pathophysiology, single-agent therapies may not be sufficient; combination therapies may also be explored. Such an approach has been effective in epilepsy, major depressive disorder, and oncology.
- The development of highly sensitive functional assessments that integrate measures of target engagement and functional activity, including the integration of digital health technologies, is essential. Functional assessment of non-motor dimensions of PD, such as cognition, may also aid in therapeutic trial planning, especially given the substantial impact of cognitive impairment and dementia on quality of life.
- A subset of individuals with abnormal αSyn levels do not phenoconvert to PD, thereby presenting a unique opportunity to identify resiliency factors that may inform novel therapeutic strategies.
- There is a critical need for animal models that accurately reflect non-motor symptoms of PD, as well as asymptomatic models of PD that capture fundamental disease biology.
- Drug target validation should involve multiple independent research groups, each operating under a single clear therapeutic hypothesis, to strengthen reproducibility and reliability.
- To better reflect heterogeneity of PD, researchers should prioritize the use of outbred mouse models rather than inbred strains. Similarly, in vitro studies should incorporate multiple iPSC lines derived from a genetically diverse cohort of PD patients.

## PD heterogeneity

PD is characterized by a broad spectrum of motor and non-motor symptoms throughout the disease course, with considerable variability in disease progression and clinical presentation among patients^5–7^. This heterogeneity underscores the need for a deeper understanding of the underlying biological mechanisms. A critical step towards achieving this goal is classifying PD into distinct subtypes based on neuropathological, genetic, and biomarker data, as well as investigating the role of neurotransmitter systems other than dopamine (e.g, cholinergic, noradrenergic and serotonergic). This approach can pave the way for precision medicine tailored to individual patient profiles^8,9^.

The complexity and overlap of those molecular mechanisms will likely determine whether a therapeutic approach targeting common factors is viable, or if a multi-target approach is necessary. Subtyping patients based solely on motor symptoms has proven unreliable^10,11^. By contrast, composite scores of motor and non-motor symptoms, biological pathology, and biomarker data have shown promise in improving diagnostic accuracy, predicting disease progression, and refining clinical trial endpoints^12^. Over the last few years, stakeholders have developed novel biological frameworks to establish diagnostic criteria based on the biological heterogeneity of underlying disease processes^13,14^. Much of the recent translational advances have focused on the development and validation of biomarkers. For example, validation of the α-synuclein (αSyn) seed amplification assay in CSF has led to the FDA qualifying it as an enrichment marker for patient stratification in clinical trials for disease-modifying therapies in neuronal synucleinopathies^12^. Furthermore, the kinetic profile of the assay carries diagnostic and prognostic weight^15^. Measurements of phosphorylated αSyn in cutaneous nerves have also provided early disease detection in synucleinopathies^16^. Finally, there are αSyn positron emission tomography (PET) tracers under development^17^.

Also discussed were some of the causes that may underly the diversity of how PD presents. These include the presence of distinct pathogenic mechanisms driven by different strains of αSyn and the contribution of co-pathologies such as Alzheimer’s disease (AD) to variability in disease manifestation. These factors should be considered during patient selection for clinical trials. αSyn pathogenesis can not only help identify patients but also address a major challenge by identifying how best to understand the disease “progression” and prediction for response to a clinical intervention. Finally, developing robust model systems that recapitulate prodromal PD, co-pathologies, non-motor symptoms, and non-dopaminergic neurotransmitter systems is imperative for advancing preclinical research and therapeutic development.

## Target validation

Although there are symptomatic treatments for PD, there are not yet any disease-modifying therapies (DMTs). While some are being pursued in clinical trials, developing DMTs remains a significant challenge^18^. Advancing candidate therapies into clinical trials requires a rigorous process of target identification and validation to ensure that the proposed target is both druggable and therapeutically relevant. It involves confirming that modulation of the target yields measurable changes in disease pathology and translates into meaningful clinical benefit^19^. Notably, recent complementary efforts have emphasized the need to systematically identify and validate promising therapeutic targets as a means to unlock therapeutics development^20^.

Several notable targets—including urate, LRRK2, TMEM175, PGK1—were discussed at the workshop, with an emphasis on the scientific and strategic considerations that influenced their validation. Integrating human genetic, pharmacological, neuroanatomic, and epidemiological data with biomarkers that demonstrate target engagement and patient enrichment has been instrumental in identifying and validating candidate drug targets. Advances in linking genetic factors to therapeutic targets have significantly improved the likelihood of regulatory approval for new treatments^21–23^. Exemplar targets such as urate, LRRK2, TMEM175, PGK1 and GPNMB continue to be a focus of the discussion as they have demonstrated direct involvement with the PD pathology and have been carefully evaluated for their druggability. The ability to safely modulate these targets raises their relevance from the therapeutics development perspective.

The most promising drug targets are supported by multiple sources of converging data. However, insufficient disease models and biomarker gaps remain key obstacles in the validation process. Before initiating target validation, researchers should determine how the target will be manipulated, identify appropriate therapeutic candidates, and determine the data necessary for robust validation. Furthermore, establishing an optimal therapeutic window based on a target’s profile is crucial to maximize the likelihood of treatment efficacy. There continues to be increasing promise in this area with the adoption of appropriate genetic models, the growth of databases and data sharing, and development of biomarkers that may track disease progression from very early stages.

## Tools to enable therapeutics development

Within industry, successful drug discovery and development programs tend to adhere to the 5Rs framework: selecting the Right target, Right patient population, Right tissue, Right safety profile, and Right commercial pathway^24^. This framework is useful in considering key tools and resources that could be essential for advancing DMTs including:

- Large and well-characterized natural history cohorts to track disease progression
- Genome-Wide Association Study or other genetic datasets to identify genetic drivers for developing PD, as well as drivers of PD progression
- Biomarkers for αSyn dysfunction, neuroinflammation, and lysosomal impairment, to facilitate patient stratification and disease monitoring
- Induced pluripotent stem cell (iPSC) lines derived from genetically diverse PD patients
- Optimized protocols for differentiating iPSCs into neuronal subtypes relevant to PD pathology
- Construct validity in mouse models for the traits of interest
- “Humanized” mouse models with PD-relevant genetic variants
- Aged mouse models with gene knockdown strategies to study late-onset disease processes
- Wild-type mouse models incorporating genetic diversity that better reflect human populations
- Genetic tools for modulating αSyn levels in non-human primates
- A database of preclinical PD models, including candidate genes and targets

Fostering a culture of open data sharing through centralized data repositories and analytical platforms is critical for accelerating research progress. Adherence to the FAIR (Findable, Accessible, Interoperable, Reusable) data principles will facilitate collaboration between academic and industry partners^25–27^. Programs such as the Linked Clinical Trials Initiative, which is a shared effort and has reviewed nearly 200 compounds since 2013, exemplifies the potential for shared efforts to streamline the translation of preclinical findings^28^. Moreover, secondary analyses of clinical trial and natural history study data may offer further opportunities for identifying and repurposing promising therapeutic candidates such as the Accelerating Medicines Partnership® in Parkinson’s Disease(AMP®-PD)^29,30^ and its second phase expanding to other related disorders^31^.

Through a concerted effort to refine disease subtyping, enhance target validation methodologies, and leverage cutting-edge research tools, the PD research community is well-positioned to advance the development of effective disease-modifying therapies.

## Biomarker use

A biomarker is a defined characteristic that is measured as an indicator of a biological process—whether physiological or pathological—or as a response to an exposure or intervention^32^. Biomarkers serve a range of critical functions and can be categorized as follows: (1) diagnostic biomarkers, which identify individuals within specific disease subtypes; (2) prognostic biomarkers, which provide insight into future clinical outcomes; (3) risk/susceptibility biomarkers, which indicate an individual’s predisposition to developing a disease; (4) disease progression (monitoring) biomarkers, which track changes in disease state over time; (5) predictive biomarkers, which inform potential therapeutic responses^32^. While the overall need for biomarkers has been known for several years, current needs and recent advances are always changing as the landscape evolves.

In the context of PD, biomarkers encompass a diverse array of modalities, including (but not limited to) fluid-based, imaging, and digital measures. Large repositories of PD fluid biomarkers have been established, and efforts are ongoing to integrate these resources to support therapeutic development^6^. Emerging technologies, such as targeted proteomic panels and digital biomarkers, enable new avenues for biomarker discovery by facilitating active behavioral tracking and passive data collection, for instance, monitoring sleep patterns^33–35^. Though data-heavy, advanced computational methods, including artificial intelligence and machine learning, can assist in the analytical and clinical validation processes.

A critical need in PD biomarker research is the development of an αSyn PET imaging biomarker, which could uniquely assess treatment efficacy or disease progression. Additional research opportunities include investigating LRRK2 protein levels, nocturnal breathing patterns, rapid eye movement (REM) sleep disturbances, neuroinflammatory pathways, lysosomal function, and quantitative molecular seed amplification levels of αSyn. Biomarkers may also flow from therapeutic targets, allowing for assessment of target engagement. Integrating multiple biomarker modalities has significant potential to capture the multifaceted nature of PD, enhancing diagnostic precision, disease monitoring, and therapeutic targeting.

## Lessons from PD adjacent communities

Communities of adjacent neurodegenerative diseases, such as AD and amyotrophic lateral sclerosis (ALS) share several similar challenges and opportunities in the pursuit of effective therapeutics. Two illustrative examples were presented at the workshop: an antibody-based approach to treat AD and a genetic approach to treat ALS.

In the AD field, an expanding repertoire of biomarkers are emerging to inform drug discovery and clinical trial design. However, the utility of biomarkers depends on their context of use (COU)—whether for assessing, disease progression, therapeutic response, or diagnostic classification in clinical trials. Biologic therapies, particularly anti-amyloid agents, have dominated recent drug development efforts in AD due to their high target specificity. But AD researchers are also investigating the potential efficacy of alternative approaches including small molecules and combination therapies. Considering the heterogeneity of PD, the most informative collection of biomarkers will likely vary depending on the COU. Furthermore, clinical outcome assessments, trial design, and statistical analyses have evolved with improved understanding of AD and the AD patient population. There is now greater emphasis on selecting appropriate clinical endpoints based on the degree of cognitive impairment within patient subgroups and genetic status, as well as tailoring data analyses to specific stakeholders—such as clinicians, patients, and healthcare payers- to enhance the interpretability and applicability of trial results. PD therapeutics development may similarly benefit from a schema to stratify patients and a collection of well-selected clinical and behavioral endpoints.

Within ALS research, there is an interest in anti-sense oligonucleotide (ASO) therapies targeting genetic mutations implicated in familial ALS, such as those in superoxide dismutase (SOD1). The SOD1 ASO, Tofersen, has received regulatory approval based on clinical efficacy, marking a significant milestone as the first therapy to improve muscle strength in a subset of patients. A key factor contributing to this success was the availability of natural history studies which facilitated early characterization of patient cohorts and optimization of therapeutic efficacy of this ASO. This strategic integration of real-world patient data undoubtedly accelerated the development of Tofersen. Likewise for PD, natural history studies that track motor and non-motor symptomatic progression may prove useful for therapeutics development.

The insights gained from the AD and ALS community underscore the importance of leveraging biomarker-driven strategies, refining clinical trial methodologies, and utilizing natural history study data to optimize patient selection and therapeutic efficacy. These lessons hold valuable implications for PD drug development, by offering a framework to enhance translational research and streamline the pathway from target discovery to clinical implementation.

## Best practices for advancing therapeutics development

Alongside insights gained from adjacent neurodegenerative communities, the PD research community can draw valuable lessons from early-stage efforts to develop PD DMTs. Two notable examples presented at the workshop were LRRK2 inhibitors and stearoyl-CoA desaturase (SCD) inhibitors.

Mechanistic understanding of how the target modulates downstream pathways of interest can enable therapeutics development. Mutations in the LRRK2 gene are associated with an increased risk of developing PD and have been linked to heightened LRRK2 activity and lysosomal dysfunction commonly observed in PD patients^36,37^. Although preclinical data support the potential of LRRK2 inhibitors as a therapeutic strategy, further research is needed to identify appropriate, target-specific biomarkers. Applying target biology to patient samples can be used to inform dose selection, optimize patient stratification, and measure treatment response in clinical trials.

SCD inhibitors target lipid dysregulation, which is influenced by both familial mutations and αSyn aggregation^38^. Given the bidirectional impact of SCD activity on lipid composition and αSyn pathology, researchers have developed a desaturation index biomarker to measure the therapeutic efficacy of SCD inhibitors. By increasing saturated fatty acids, these inhibitors can stabilize lipid membrane interactions by reducing αSyn aggregation and its associated toxicity. Further research is ongoing to explain the relationship between SCD inhibition and the expected amount of target modulation that would be needed to see a clinical benefit in humans.

Biomarkers that are predictive of efficacy are needed to accelerate PD therapeutics development overall. As described in the above section, the field is working on developing and validating both direct biomarkers of αSyn aggregation and sensitive measures of motor function and other phenotypic outcomes, including digital endpoints. Innovations in PD clinical development (including biomarkers for early diagnosis of patients, biomarkers for efficient decision-making and sensitive endpoints are necessary to shorten clinical development timelines.

Despite an improving landscape, PD drug development continues to face significant challenges. These include the lack of a universal surrogate endpoint for accelerating drug approvals and a standardized framework to evaluate disease progression, contributing to failures in clinical trials and hindering the translation of promising therapeutic candidates into approved treatments. Notably, ongoing advancements in both research areas are underway to drive the process forward, potentially towards precision medicine approaches^13,14,39–41^.

To address some of the barriers in therapeutics development, SPARK NS has launched a breakthrough initiative aimed at bridging the gap between academia and industry. The program provides funding, education, mentorship, and networking opportunities to support researchers in advancing therapeutic candidates from preclinical development to clinical trials^42^. By equipping investigators with the necessary resources and infrastructure, this initiative fosters interdisciplinary collaboration and enhances the likelihood of clinical trial success.

## Concluding thoughts

The two-day workshop yielded several insights into existing gaps in PD research and identified strategies for enhancing therapeutic development through a collaborative interdisciplinary approach. Though not an exhaustive list, several cross-cutting themes emerged during the discussions, highlighting areas where collective efforts may drive meaningful progress.

### Collaboration

Advancing PD therapeutics requires a concerted effort among academic researchers, industry partners, independent investigators, regulatory agencies, non-profit organizations and PWLEs. Strengthening these collaborations will foster a more robust therapeutic development ecosystem, accelerate both preclinical and clinical development, and support the training of the next generation of researchers. Additionally, the field should work towards building a shared infrastructure that enables querying and analysis of large datasets while also prioritizing patient engagement and education about clinical trial results.

### Fundamental research

A deeper mechanistic understanding of non-motor symptoms in PD such as cognitive deficits remains a critical unmet need which requires additional focus to pursue therapeutics that address these symptoms. While continued advancements in tools, such as novel animal models are essential, emphasis should be placed on prioritizing using these tools to identify and validate robust therapeutic targets. Moreover, a biologically driven approach to understanding PD heterogeneity is imperative to inform personalized treatment strategies.

### Enabling technologies

Establishing sensitive and reliable functional endpoints in clinical trials is essential for evaluating therapeutic efficacy and understanding disease progression. Equally important is the development and validation of biomarkers that can facilitate the biological subtyping of PD patients and serve as reliable indicators of disease progression and treatment response. While PD animal models may not fully recapitulate all behavioral phenotypes, they remain valuable for optimizing therapeutic dosing and elucidating disease mechanisms at both cellular and organismal levels.

### Future outlook

In the PD space, the PWLE are engaged and committed. Furthermore, there is a strong sense of community within the advocacy groups. Finally, advances in basic science are helping identify and validate new targets. Considering this composite current state of the field and the willingness to collaborate, there are reasons to be optimistic about the road ahead for PD therapeutics development.

## Supplementary information

Supplementary Information

## Data Availability

No datasets were generated or analysed during the current study.
