# Supplementary material for: Mapping the developmental path for Parkinson’s disease therapeutics
Source: NPJ Parkinsons Dis. 2025 Nov 7;11:313. doi: 10.1038/s41531-025-01154-1 (PMC12594996; doi:10.1038/s41531-025-01154-1)
Supplement: Supplementary file 1 — Supplementary Information [file 41531_2025_1154_MOESM1_ESM.pdf]

Mapping the Developmental Path for Parkinson's Disease Therapeutics

April 23–24, 2024

Authors: Neel T. Dhruv[1*], Sarah Robinson Schwartz[1], Christine Swanson-Fischer[1], Hyun Joo Cho[1], Rebekah Corlew[1], Lyn Jakeman[1], Lauren A. Laboissonniere[1], Rebecca Price[1], Shireen Sarraf[1], Beth-Anne Sieber[1], Christine Torborg[1], Carl Wonders[1], Alice Chen-Plotkin[2], William J. Martin[3], Amir P. Tamiz[1*]

[1] National Institute of Neurological Disorders and Stroke, Bethesda, MD, USA
[2] Department of Neurology, Perelman School of Medicine, University of Pennsylvania, Philadelphia, PA, USA.
[3] Johnson & Johnson, Inc., San Diego, CA, USA
[*] Corresponding authors: neel.dhruv@nih.gov; amir.tamiz@nih.gov

Supplementary Information: Agenda

April 23, 2024 (Day 1)

All times Eastern

Welcome and Opening remarks

9:00 am **NINDS Welcome**
Walter J. Koroshetz, MD
Director, National Institute of Neurological Disorders and Stroke

9:10 am **Engaging the lived experience perspective on PD Therapeutics**
Rebekah Corlew, PhD
Lived Experience Strategist, National Institute of Neurological Disorders and Stroke

Keynote Address: Landscape of PD therapeutics development

9:20 am **Workshop Co-Chairs welcome and goals for the 2 days**
Alice Chen-Plotkin, MD
Parker Family Professor of Neurology, University of Pennsylvania

Bill Martin, PhD
Global Neuroscience Therapeutic Area Head, Johnson & Johnson Innovative Medicine

10:00 am **Break (5 minutes)**

Session 1: PD Heterogeneity

Moderator: Karl Kieburtz, MD, MPH, Robert J. Joynt Professor in Neurology, University of Rochester School of Medicine and Dentistry

10:05 am **Motor Heterogeneity in Parkinson's Disease: Challenges to Therapeutic Developments**
Anthony Lang, MD, FRCPC,
Professor, Department of Medicine, University of Toronto

10:20 am **Heterogeneity of Non-Motor Symptoms across Lewy Body Diseases**
Kathleen Poston, MD, MS,
Professor of Neurology & Neurological Sciences, Stanford University

10:35 am **Q & A discussion (30 minutes)**

11:05 am **Break (10 minutes)**

Session 2: Target validation

Moderator: Alice Chen-Plotkin, MD, Parker Family Professor of Neurology, University of Pennsylvania

11:15 am **Evolving Arcs toward Disease-Modifying Therapy for Parkinson's**
Michael Schwarzschild, MD, PhD
Professor of Neurology, Massachusetts General Hospital

11:30 am **Targeting LRRK2: Why, Who, Where and When**
Mark R Cookson, PhD
Senior Investigator and Chief, Laboratory of Neurogenetics
National Institute on Aging

11:45 am **An Industry Perspective on Target Validation For Neurologic Diseases**
David Stone, PhD
VP, Head of Genetics and Target Identification, Cerevel Therapeutics, LLC

12:00 pm **Q & A discussion (30 minutes)**

12:30 pm **Lunch (60 minutes)**

Session 3: Appropriate tools to enable therapeutics development

Moderator: Shalini Padmanabhan, PhD, Vice President, Discovery & Translational Research, Michael J. Fox Foundation

1:30 pm **Teeing Up a Discussion about Appropriate Tools to Enable Therapeutics Development**
Patrik Brundin, MD, PhD
Therapeutic Area Leader for Movement Disorders, Roche

1:45 pm **Preclinical Tools to Advance Parkinson Disease Therapeutics: What They Can and Cannot Do**
Laura Volpicelli-Daley, PhD
Associate Professor Parkinson Association of Alabama Professorship, University of Alabama at Birmingham

2:00 pm **Q & A discussion (30 minutes)**

2:30 pm **Break (10 minutes)**

Session 4: Biomarker use

Moderator: Danielle Graham, PhD, Head of Fluid Biomarkers, Biogen

2:40 pm **AI-Enabled Biomarkers: A Window into Parkinson's Progression, Detection, and Treatment Response**
Dina Katabi, PhD
Thuan and Nicole Pham Professor and MacArthur Fellow, Massachusetts Institute of Technology

2:55 pm **Identification of Proteomic Targets and Biomarkers: The Mapping Proteomics to Parkinson's Disease Project (MAP2PD)**
Laura Winchester, DPhil
Alzheimer's Research UK Fellow, University of Oxford

3:10 pm **Understanding Biomarker Parameters to Enable Clinical Trials in Parkinson's Disease**
Danielle Graham, PhD
Head of Fluid Biomarkers, Biogen

3:25 pm **Q & A discussion (30 minutes)**

3:55 pm **Break (15 minutes)**

Breakout Session Day 1

4:10 pm **Parallel breakout session**
What is missing that is holding back therapeutics development?

4:55 pm **Break (5 minutes)**

End of Day 1

5:00 pm Day 1 wrap-up

5:10 pm **Adjourn for the day**

April 24, 2024 (Day 2)

All times Eastern

Welcome and Day 2 Opening remarks

8:45 am **Workshop Co-Chairs Welcome**
Alice Chen-Plotkin, MD
Parker Family Professor of Neurology, University of Pennsylvania

Bill Martin, PhD
Global Neuroscience Therapeutic Area Head, Johnson & Johnson Innovative Medicine

Breakout Reports from Day 1

8:55 am **Reports from Breakout sessions and Q & A**
Breakout Reports by the breakout session leaders followed by general discussion.

10:00 am **Break (10 minutes)**

Session 5: Lessons from PD adjacent communities

Moderator: Amir Tamiz, PhD, Director, Division of Translational Research, National Institute of Neurological Disorders and Stroke

10:10 am **Disease-Modifying Therapies: Lessons from Alzheimer's Disease Drug Development**
Jeffrey Cummings, MD, ScD
Research Professor, Department of Brain Health, University of Nevada, Las Vegas

10:25 am **RNA-Target Therapies for Neurodegenerative Disease**
Timothy M. Miller, MD, PhD
David Clayson Professor of Neurology, Washington University School of Medicine

10:40 am **Q & A discussion (30 minutes)**

11:10 am **Break (10 minutes)**

Session 6: Best practices for advancing development

Moderator: Bill Martin, PhD, Global Neuroscience Therapeutic Area Head, Johnson & Johnson Innovative Medicine

11:20 am **Addressing the Academia-Industry Gap to Advance Discoveries to the Clinic**
Opher Kornfeld, PhD
Director, Translational Research and Development, SPARK NS

11:35 am **LRRK2 Inhibitors: From Basic Biology to the Clinic**
Anastasia Henry, PhD
Director and Principal Scientist, Denali Therapeutics

11:50 am **Opportunities and Challenges in Parkinson's Disease Clinical Development using the Example of the SCD Inhibitor YTX-7739**
Fiona Elwood, PhD
VP, Disease Area Leader, Neurodegeneration, Neuroscience, Johnson & Johnson

12:05 pm **Q & A discussion (30 minutes)**

End of Day 2

12:35 pm Day 2 wrap-up

12:45 pm **Adjourn / Lunch (75 minutes)**

Bonus Session Day 2

2:00 pm **Parallel sessions on focused topics**

- **What to know about IP and patents** *with Lauren Nguyen-Antczak, PhD,* Senior Licensing & Patenting Manager, National Cancer Institute and *Susan Ano, PhD,* Director, Office of Technology Transfer, NINDS
- **Forming partnerships with industry** *with Bill Martin, Patrik Brundin, and John Sullivan, PhD,* Entrepreneur in Residence, SEED, NIH
- **Starting a company** *with Opher Kornfeld and Leon Chen, PhD*, Partner at The Column Group

3:00 pm **Adjourn**
